# Inhibition of Shp2 suppresses mutant EGFR-induced lung tumors in transgenic mouse model of lung adenocarcinoma

**DOI:** 10.18632/oncotarget.3356

**Published:** 2015-01-31

**Authors:** Valentina E. Schneeberger, Yuan Ren, Noreen Luetteke, Qingling Huang, Liwei Chen, Harshani R. Lawrence, Nicholas J. Lawrence, Eric B. Haura, John M. Koomen, Domenico Coppola, Jie Wu

**Affiliations:** ^1^ Department of Molecular Oncology, H. Lee Moffitt Cancer Center and Research Institute, Tampa, Florida, USA; ^2^ Division of Cell Biology, Microbiology, and Molecular Biology, University of South Florida, Tampa, Florida, USA; ^3^ Small Animal Modeling and Imaging Core, H. Lee Moffitt Cancer Center and Research Institute, Tampa, Florida, USA; ^4^ Department of Drug Discovery, H. Lee Moffitt Cancer Center and Research Institute, Tampa, Florida, USA; ^5^ Department of Oncologic Sciences, University of South Florida College of Medicine, Tampa, Florida, USA; ^6^ Department of Thoracic Oncology, H. Lee Moffitt Cancer Center and Research Institute, Tampa, Florida, USA; ^7^ Department of Anatomic Pathology, H. Lee Moffitt Cancer Center and Research Institute, Tampa, Florida, USA

**Keywords:** Shp2, EGFR, phosphatase, transgenic mice, lung cancer

## Abstract

Epidermal growth factor receptor (EGFR) mutants drive lung tumorigenesis and are targeted for therapy. However, resistance to EGFR inhibitors has been observed, in which the mutant EGFR remains active. Thus, it is important to uncover mediators of EGFR mutant-driven lung tumors to develop new treatment strategies. The protein tyrosine phosphatase (PTP) Shp2 mediates EGF signaling. Nevertheless, it is unclear if Shp2 is activated by oncogenic EGFR mutants in lung carcinoma or if inhibiting the Shp2 PTP activity can suppress EGFR mutant-induced lung adenocarcinoma. Here, we generated transgenic mice containing a doxycycline (Dox)-inducible PTP-defective Shp2 mutant (tetO-Shp2CSDA). Using the rat Clara cell secretory protein (CCSP)-rtTA-directed transgene expression in the type II lung pneumocytes of transgenic mice, we found that the Gab1-Shp2 pathway was activated by EGFR^L858R^ in the lungs of transgenic mice. Consistently, the Gab1-Shp2 pathway was activated in human lung adenocarcinoma cells containing mutant EGFR. Importantly, Shp2CSDA inhibited EGFR^L858R^-induced lung adenocarcinoma in transgenic animals. Analysis of lung tissues showed that Shp2CSDA suppressed Gab1 tyrosine phosphorylation and Gab1-Shp2 association, suggesting that Shp2 modulates a positive feedback loop to regulate its own activity. These results show that inhibition of the Shp2 PTP activity impairs mutant EGFR signaling and suppresses EGFR^L858R^-driven lung adenocarcinoma.

## INTRODUCTION

Shp2 is a nonreceptor PTP encoded by the human *PTPN11* gene [1]. It has tandem SH2 domains in the N-terminal region, a PTP domain, and a C-terminal region containing tyrosine phosphorylation sites. Binding of Shp2 SH2 domains to specific tyrosine phosphorylated sites relieves autoinhibition and activates Shp2. In epidermal growth factor (EGF)-stimulated cells, Shp2 binds to tyrosine-phosphorylated Gab1 at the bisphosphoryl tyrosine-based activation motif (BTAM) consisting of phosphorylated Tyr-627 and Tyr-659 [2]. Gab1-Shp2 binding activates the Shp2 PTP activity and mediates activation of Erk1/2 and Src family kinases (SFKs) by EGF [2-5]. Thus, in addition to EGFR, EGF paradoxically activates a PTP to mediate the EGFR protein tyrosine kinase (PTK) signaling.

Knockdown of Shp2 by shRNAs partially inhibits proliferation of cancer cells in cell cultures [6]. Importantly, far greater effects of Shp2 knockdown have been observed consistently in tumor xenograft growth assays *in vivo*, suggesting that Shp2 plays a critical role in tumor growth [6, 7]. Since Shp2 plays a positive role in oncogenic signaling and tumorigenesis, it is a potential target for development of novel anti-cancer drugs. In fact, several efforts are underway to develop Shp2 inhibitors as potential therapeutic agents [8-14].

*EGFR* is the second most frequently mutated oncogene in lung adenocarcinoma after *KRAS* [15]. Significantly, Shp2 is a positive regulator of both EGFR and Ras signaling. Moreover, gain-of-function (GOF) Shp2 mutants are found in human lung carcinomas and can induce lung tumors in mice [16, 17]. Approximately 80% of EGFR mutations in non-small cell lung cancer (NSCLC) are either deletion of the conserved four amino acids LREA residues in exon 19 or a L858R point mutation in exon 21 [18]. Expression of these GOF EGFR mutants in type II lung pneumocytes directed by a rat Clara cell secretory protein (CCSP) promoter in CCSP-rtTA/tetO-EGFR mutant bitransgenic mice induces lung adenocarcinoma [19-21]. NSCLC harboring these GOF EGFR PTK domain mutants are selectively sensitive to the EGFR-selective PTK inhibitors (TKIs) erlotinib and gefitinib. However, *de novo* and acquired drug resistance mechanisms such as the gatekeeper T790M EGFR mutation have been observed in lung cancer patients [18, 21, 22]. Therefore, it is necessary to develop new EGFR PTK inhibitors and/or to target additional tumor promoting molecules to improve lung cancer treatment [18, 21, 22].

Although EGF stimulates Shp2 activation, it is not entirely clear whether Shp2 is active in lung epithelial cells harboring GOF EGFR mutants and whether Shp2 is important for mutant EGFR to drive lung adenocarcinoma. In this study, we generated transgenic mice expressing a PTP-defective (catalytic residues C459S/D425A mutations), dominant-negative Shp2 mutant (tetO-Shp2CSDA) to assess the effects of Shp2 PTP inhibition in a transgenic mouse model of mutant EGFR-driven lung adenocarcinoma. Using NSCLC cell lines carrying GOF EGFR mutants and transgenic mice expressing EGFR^L858R^, we provide evidence that EGFR mutants activate Shp2 in human lung adenocarcinoma cells and in mouse lung tissues. Furthermore, Shp2CSDA suppresses EGFR^L858R^-induced lung adenocarcinoma in transgenic animals.

## RESULTS

### Shp2 signaling pathway is activated by mutant EGFR in lung adenocarcinoma cells

**Figure 1:**
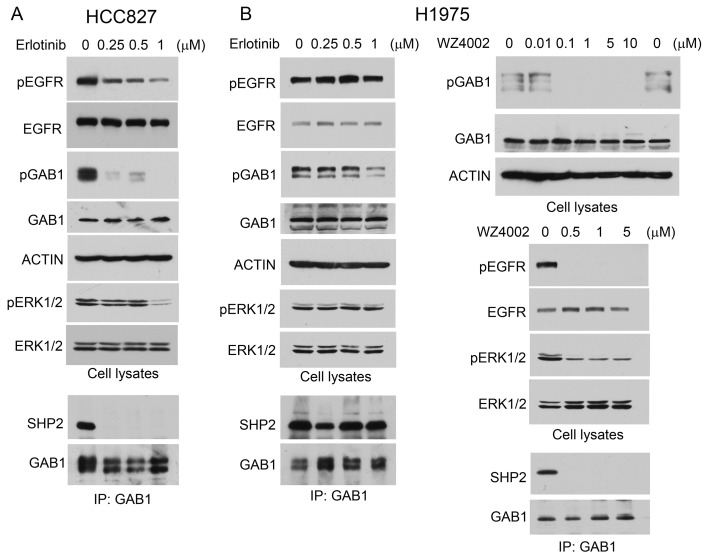
Shp2-mediated Erk1/2 pathway is activated by mutant EGFR in lung adenocarcinoma cells HCC827 (A) and H1975 (B) cells were mock-treated or treated with EGFR PTK inhibitors erlotinib or WZ4002 as indicated. Cell lysates were analyzed by immunoblotting with indicated antibodies or subjected to immunoprecipitation with anti-Gab1 antibody and immunoprecipitates were analyzed by immunoblotting with antibodies to Shp2 or Gab1.

EGFR activates Shp2 by phosphorylating Gab1, which binds and activates Shp2 [2]. In HCC827 and H1975 human lung adenocarcinoma cells that harbor mutant EGFR (del19 and L858R/T790M mutations, respectively), Gab1 was constitutively tyrosine phosphorylated and bound Shp2 (Fig. 1). This indicates that Shp2 is constitutively activated in these lung adenocarcinoma cells. Moreover, active Erk1/2 (pErk1/2) was readily detectable in these cells (Fig. 1). To determine whether Gab1 tyrosine phosphorylation and binding to Shp2 are attributed to mutant EGFR in these cells, we treated HCC827 and H1975 cells with the EGFR tyrosine kinase inhibitor erlotinib or WZ4002. Erlotinib inhibited EGFR and Gab1 tyrosine phosphorylation in HCC827 cells at the lowest concentration tested (0.25 μM). This led to dissociation of Shp2 from Gab1 (Fig. 1A). H1975 cells are resistant to erlotinib due to the T790M gatekeeper mutation [21]. Hence, erlotinib did not cause Gab1-Shp2 dissociation in H1975 cells (Fig. 1B). WZ4002 was reported to inhibit the EGFR T790M mutant [23]. Treatment of H1975 cells with WZ4002 inhibited EGFR and Gab1 tyrosine phosphorylation and resulted in Gab1-Shp2 dissociation (Fig. 1B, right panels).

An established role of Shp2 in EGFR signaling is to mediate Erk1/2 activation. As shown in Fig. 1, inhibition of Gab1-Shp2 interaction by erlotinib in HCC827 and by WZ4002 in H1975 cells correlated with inactivation of Erk1/2. In comparison, erlotinib did not inhibit Erk1/2 in H1975 cells. These data indicate that the Shp2 signaling pathway is activated by the GOF EGFR mutants in these lung adenocarcinoma cells.

**Figure 2:**
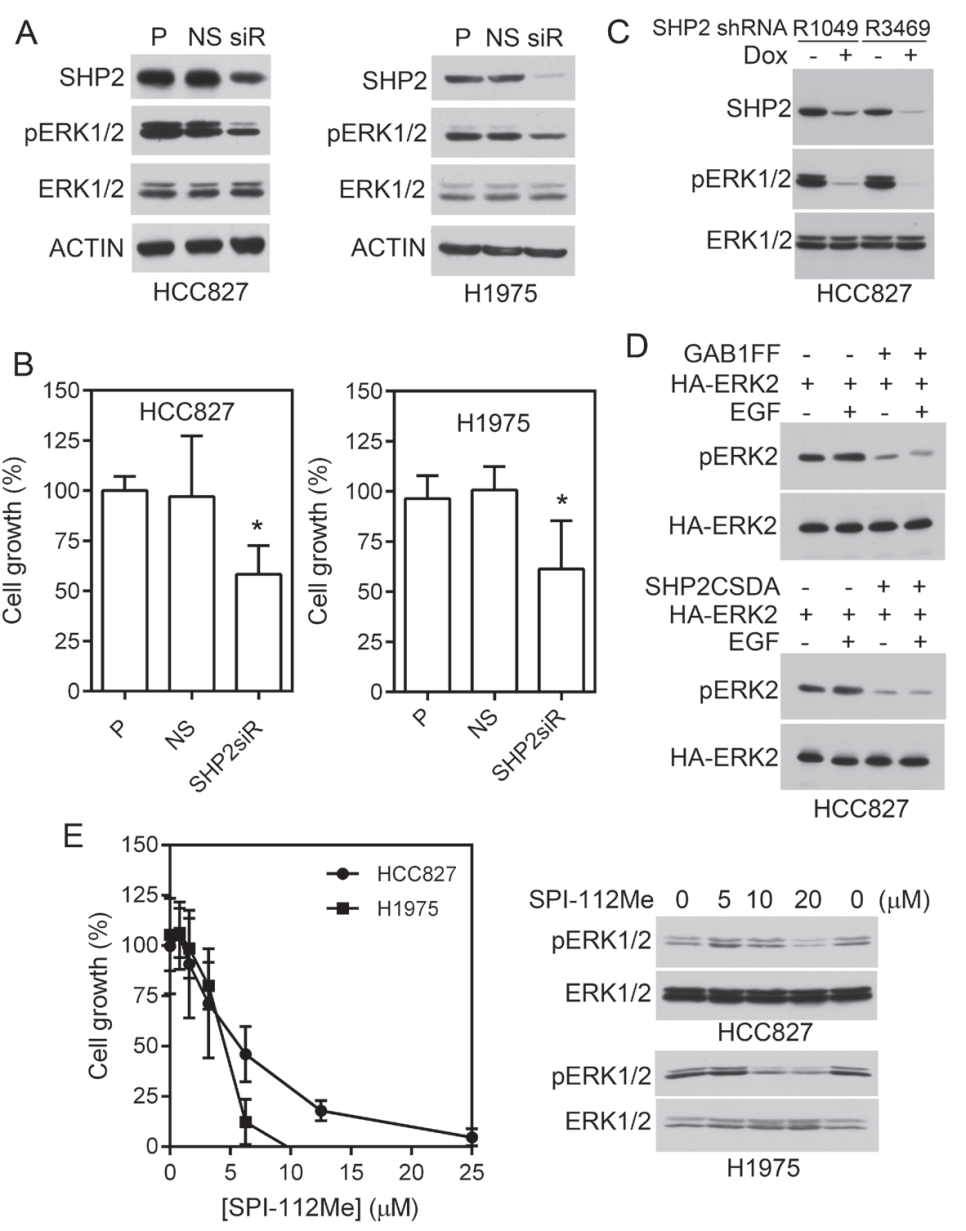
Effects of Shp2 inhibition on lung adenocarcinoma cells containing mutant EGFR A, HCC827 and H1975 cells were transfected with Shp2 siRNAs (siR), non-silencing control siRNA (NS), or mock-treated (P). Cell lysates were analyzed for Shp2 knockdown and active Erk1/2 by immunoblotting. B, Cells were plated in 96-well plates followed by transfection with Shp2 siRNAs or control siRNA as above. The relative number of viable cells was measured 6 days post transfection and normalized to untransfected cells. Data represent two (HCC827) and four (H1975) independent experiments performed in quintuple. *, *p* <0.05. C, HCC827 containing Dox-inducible Shp2 shRNAs [6] were treated with Dox or left untreated, cell lysates were analyzed with indicated antibodies. D, HCC827 cells were transfected with HA-Erk2 plus control vector (−), Gab1FF, or Shp2CSDA, and treated with EGF or left untreated. HA-Erk2 activation were analyzed by immunoblotting of pErk2 after immunoprecipitation of the transfected HA-Erk2. E, HCC827 and H1975 cells were treated with indicated concentrations of SPI-112Me in 96-well plates as described in Materials and Methods and viable cells were measured on Day 6. Data were from three independent experiments performed in triplicates (n = 9) (graph). Right panel, cells were treated with SPI-112Me overnight and cell lysates were analyzed by immunoblots with indicated antibodies.

To determine if Shp2 is involved in cell proliferation of HCC827 and H1975 cells, we used siRNAs to knock down Shp2 in these cells. Consistent with a role of Shp2 in mediating Erk1/2 activation, Shp2 knockdown reduced pErk1/2 level in HCC827 and H1975 cells (Fig. 2A). While the non-silencing (NS) siRNAs had no effect on the pErk1/2 level or cell proliferation, Shp2 knockdown inhibited proliferation of HCC827 and H1975 cells by 42% and 39%, respectively (Fig. 2A, B).

To further confirm that Shp2, Gab1-Shp2 interaction, and Shp2 PTP activity mediate Erk1/2 activation in lung adenocarcinoma cells that harbor mutant EGFR, we compared the pErk1/2 level in isogenic HCC827 cells containing two different Dox-inducible Shp2 shRNAs (Fig. 2C). We also determined effects of a Shp2-binding defective Gab1Y627F/Y659F mutant (Gab1FF) and a Shp2 PTP-inactive mutant (Shp2CSDA) on Erk1/2 activation in HCC827 cells (Fig. 2D). The pErk1/2 level was markedly reduced in Dox-induced Shp2 knockdown in HCC827 (Fig. 2C). Expression of Gab1FF or Shp2CSDA inhibited activation of co-transfected Erk2 both in the absence or presence of EGF exposure in HCC827 cells (Fig. 2D). These data demonstrate that Gab1-Shp2 interaction and Shp2 PTP activity play important roles in mediating Erk1/2 activation by the mutant EGFR in HCC827 cells.

SPI-112Me is a cell permeable Shp2 inhibitor [11, 24]. To further evaluate the role of Shp2 PTP activity in HCC827 and H1975 cells, we treated these cells with various concentrations of SPI-112Me. SPI-112Me inhibited pErk1/2 and proliferation of HCC827 and H1975 cells (Fig. 2E).

### Generation of transgenic mice carrying a Dox-inducible PTP-defective Shp2 mutant

**Figure 3:**
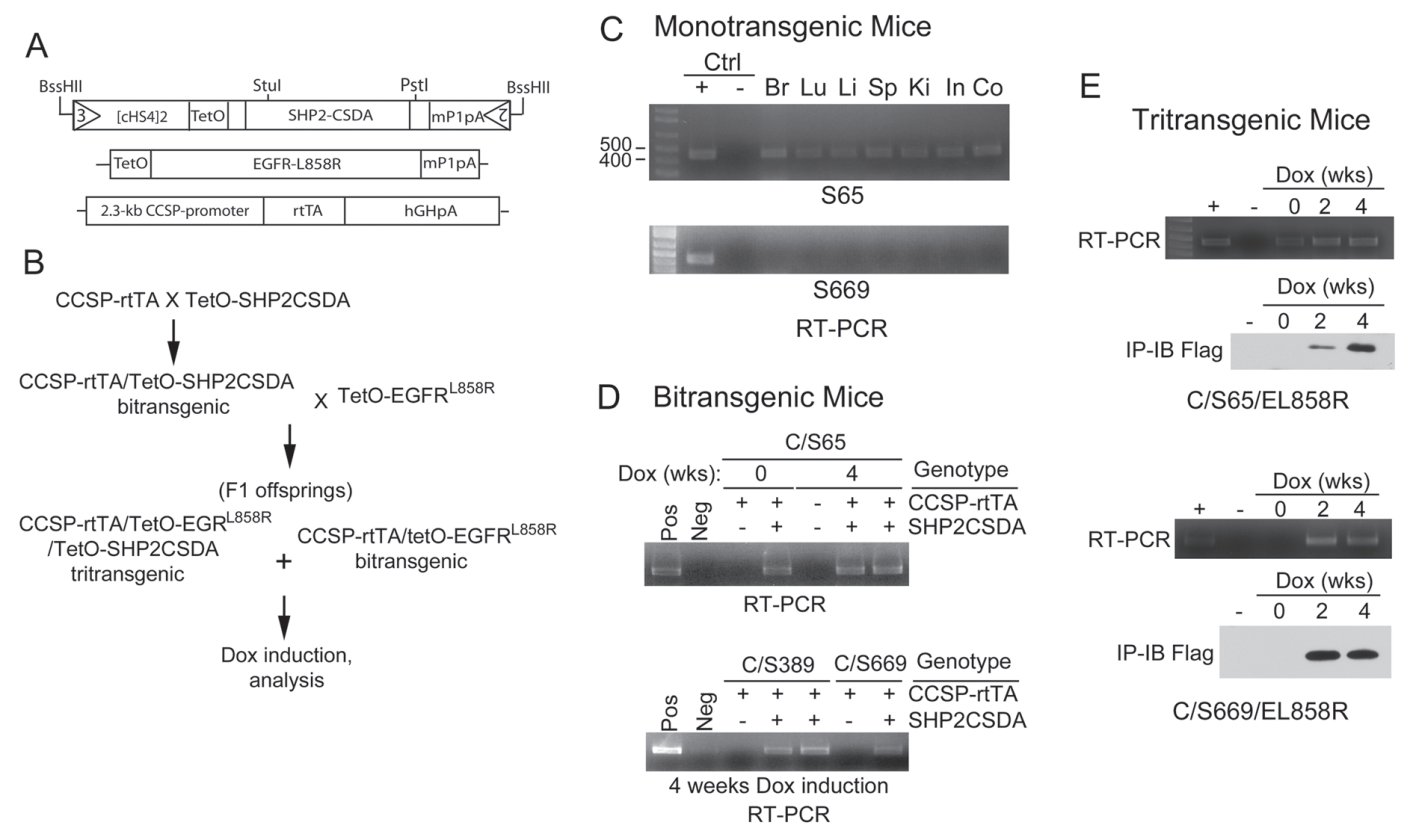
Generation and characterization of transgenic mice A, Diagrams of the tetO-Shp2CSDA, tetO-EGFR^L858R^ [19] and CCSP-rtTA [25] transgenes of mice used in this study. B, The breeding scheme for production of bitransgenic and tritransgenic mice. C, Tissue samples from brain (Br), lung (Lu), liver (Li), spleen (Sp), kidney (Ki), small intestine (In), and colon (Co) of monotransgenic tetO-Shp2CSDA Line-65 (S65) and Line-669 (S669) mice were analyzed by RT-PCR for the presence of Shp2CSDA mRNA. D, C/Sdn bitransgenic mice from Line-65, Line-389, and Line-669 were fed with regular chow or Dox diet for 4 weeks. Lung tissue samples were analyzed by RT-PCR to examine Shp2CSDA mRNA expression. E, C/S65/EL858R and C/S669/EL858R tritransgenic mice were fed with regular rodent chow or Dox diet for 2 or 4 weeks. Lung tissue samples were analyzed by RT-PCR for mRNA expression or by immunoprecipitation-immunoblotting analysis for the presence of Shp2CSDA protein.

GOF EGFR mutants induce lung tumors in transgenic mouse models of lung adenocarcinoma [19-21]. To determine if inhibition of Shp2 PTP activity can suppress lung tumor development in a transgenic mouse model of mutant EGFR, we generated transgenic mice containing a Dox-inducible PTP-defective, dominant-negative Shp2 (Shp2CSDA). By design, controlled expression of Shp2CSDA in the progenitor cells of lung adenocarcinoma can be achieved by breeding the tetO-Shp2CSDA transgenic mice with CCSP-rtTA transgenic mice [25] and feeding the CCSP-rtTA/tetO-Shp2CSDA (C/Sdn) bitransgenic mice with Dox diet (Fig. 3B).

We obtained 41 pups from two separate microinjection experiments. Among them, 9 founder lines exhibited germline transmission of the tetO-Shp2CSDA transgene. By RT-PCR analysis of mRNA expression and immunoprecipitation-immunoblotting analysis of protein expression, we identified transgenic mice that either had detectable leaky (un-induced) expression of the Shp2CSDA transgene or had no detectable leaky expression (Fig. 3 and Supplementary Fig. 1). Some of these transgenic lines were crossed with CCSP-rtTA mice to generate C/Sdn bitransgenic mice and screened for Dox-inducible expression of Shp2CSDA in the lung. After initial characterization, we selected Line-65 and Line-669 for the subsequent study (Fig. 3). Line-65 had detectable mRNA of the Shp2CSDA transgene in the absence of Dox in the brain, lung, liver, spleen, kidney, small and large intestine. Nevertheless, upon Dox induction, Shp2CSDA expression was increased in the lung of these mice (Fig. 3E). Line-669, as well as Line-389, had no detectable Shp2CSDA in the absence of Dox and showed Dox-induced expression of Shp2CSDA mRNA and protein in the lung. Moreover, Dox-induced bi- or tritransgenic mice containing Line-65 did not appear to have higher Shp2CSDA protein in the lung tissues than those containing Line-669.

Although Line-65 mice have a low level of leaky expression of the Shp2CSDA mRNA, like other Shp2CSDA transgenic mouse lines generated in our study, we have not observed any abnormal growth or breeding behavior. Histology of lungs from Dox-fed bitransgenic C/Sdn mice was indistinguishable from that of the wildtype mice (Supplementary Fig. 2).

### Shp2CSDA suppresses EGFR^L858R^-induced Erk1/2 and Src activation

To determine if the PTP-defective Shp2CSDA inhibits Shp2-mediated EGFR signaling in the lungs of transgenic mice, we produced CCSP-rtTA/tetO-EGFR^L858R^ (C/EL858R) bitransgenic mice and CCSP-rtTA/tetO-Shp2CSDA/tetO-EGFR^L858R^ (C/Sdn/EL858R) tritransgenic mice. This was accomplished by crossing C/Sdn bitransgenic mice with tetO-EGFR^L858R^ mice (Fig. 3B). The resulting F1 offspring were then used in our study. After induction with Dox for 2-8 weeks, lung tissues from the transgenic mice were analyzed.

**Figure 4:**
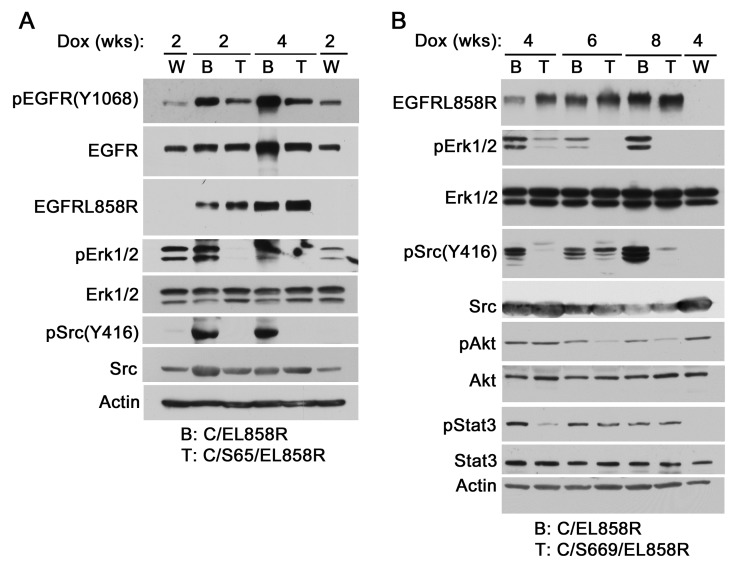
EGFR^L858R^-activated signaling molecules in transgenic mice Transgenic mice were fed with Dox diet for 2-8 weeks. Lung tissues were flushed twice with 10 ml PBS and snap-frozen in liquid nitrogen. Frozen tissues were thawed and lysed with the lysis buffer. Equal amount of proteins in Triton X-100 soluble supernatants were analyzed by immunoblotting with indicated antibodies. W, wildtype, B, C/EL858R bitransgenic, T, C/S65/EL858R or C/S669/EL858R tritransgenic.

Immunoblot analysis using an EGFR^L858R^-specific antibody confirmed Dox-induced expression of the EGFR^L858R^ mutant in C/EL858R bitransgenic mice and C/Sdn/EL858R tritransgenic mice (Fig. 4). Compared to wildtype control mice, pErk1/2 and pSrc(Y416) levels were increased in the lungs of Dox-induced C/EL858R bitransgenic mice, indicating that EGFR^L858R^ activated Erk1/2 and Src in the lungs of these mice. In 8 of 9 lung tissue samples that we have analyzed, the pErk1/2 levels were decreased in the Dox-induced C/Sdn/EL858R tritransgenic mice compared to the C/EL858R bitransgenic mice (Fig. 4 and Supplementary Fig. 3). Similarly, pSrc(Y416) levels were decreased in 5 of 5 lung tissue samples from the Dox-induced tritransgenic mice compared to the samples from the C/EL858R bitransgenic mice. In contrast, no consistent effects on pAkt and pStat3 were observed in the Dox-induced tritransgenic mice (Fig. 4 and Supplementary Fig. 3). These results suggest that Shp2CSDA inhibited EGFR^L858R^-induced Erk1/2 and Src activation in the lungs of these tritransgenic mice.

### Shp2CSDA inhibits EGFR^L858R^-induced lung tumors

**Figure 5:**
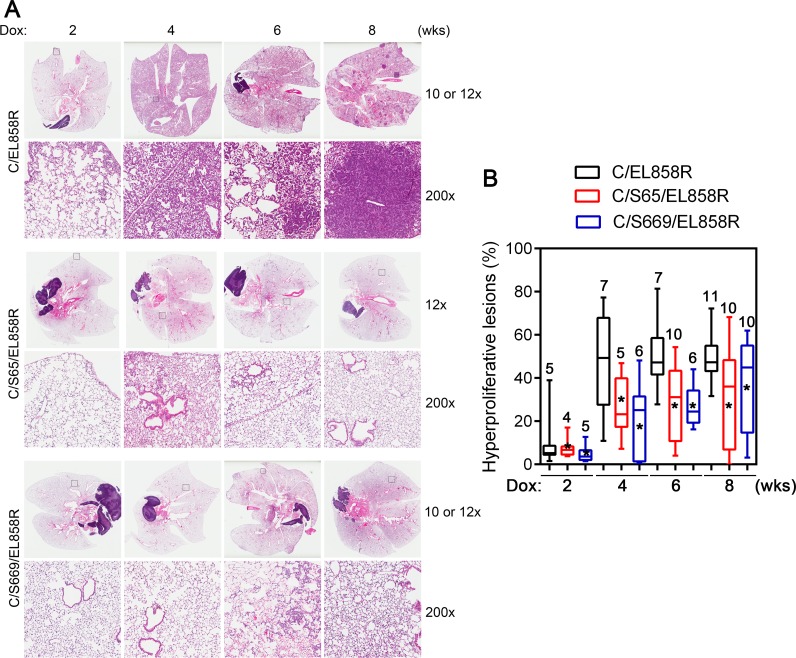
Lung hyperproliferative lesions in transgenic mice A, H&E sections of lungs from transgenic mice. Transgenic mice were fed with Dox diet for 2-8 weeks. Lungs were collected and processed as described in Materials and Methods. H&E stained slides were scanned with a ScanScope XT slide scanner (Aperio). Examples of H&E stained sections at each time point after Dox induction from bitransgenic and two different tritransgenic lines are presented. Top panels from each transgenic line show images of whole lung sections. The bottom panels from each transgenic line show a higher magnification (200x) of the boxed areas. B, Box-and-Whiskers plot of the extent of hyperproliferative lesions in the lungs of Dox-induced transgenic mice. Three 4 μm sections that were 25 μm apart from each lung paraffin block were stained with H&E. Images were acquired and analyzed using a histology pattern recognition algorithm (Aperio). Areas of hyperproliferative lesions were calculated. Numbers on each column indicate the number of mice analyzed in that set of samples. *, *p* <0.05. Hyperproliferative lesions include atypical adenomatous hyperplasia, non-mucinous adenocarcinoma with lepidic pattern, bronchioloalveolar adenoma, and solid adenoma (<0.5 mm in diameter) and adenocarcinoma (>0.5 mm in diameter).

To analyze the effects of Shp2CSDA on EGFR^L858R^-induced lung tumors, C/EL858R and C/Sdn/EL858R mice were induced with Dox for 2 to 8 weeks and lung tissues were stained with H&E for histological examination. Within 2 weeks of Dox induction, C/EL858R mice developed focal atypical adenomatous hyperplasia (Fig. 5A). This progressed rapidly to extensive, diffused non-mucinous adenocarcinoma with lepidic pattern within 4 weeks after Dox-induction (Fig. 5A, second column). Bronchioloalveolar adenoma appeared at week 6 and progressed to more solid adenoma and adenocarcinoma (> 0.5 mm in diameter) by week 8 (Fig. 5A). In comparison, following parallel Dox induction, C/Sdn/EL858R tritransgenic mice from both Line-65 and Line-669 developed markedly fewer hyperproliferative lesions compared to C/EL858R bitransgenic mice (Fig. 5A, B).

To measure the diffuse lung hyperproliferative lesions in these mouse lung tissues, we used the Aperio Genie® histology pattern recognition program to semi-quantitatively estimate areas of hyperproliferative lesions as described in Materials and Methods (Supplementary Fig. 4). Although the algorithm recognized the small bronchiolar epithelia as lesions, this systematical error did not affect our comparison of lung hyperproliferative lesions because it generally affected <1% of the estimated lesion areas and all lung H&E slides were measured using the same set of parameters.

Using this semi-quantitative measurement, the average areas of lung hyperproliferative lesions were 8.2, 50.2, 51.2, and 49.5% in C/EL858R bitransgenic mice induced with Dox for 2, 4, 6, and 8 weeks, respectively (Fig. 5B). In comparison, the average areas of lung hyperproliferative lesions in Line-65 of C/Sdn/EL858R (C/S65/EL858R) tritransgenic mice were 7.1, 26.8, 28.9, and 31.3% after induction with Dox for 2, 4, 6, and 8 weeks, respectively. In the Line-669 of C/Sdn/EL858R (C/S669/EL858R) tritransgenic mice, the average areas of lung hyperproliferative lesions after the same time periods of induction were 4.7, 21.4, 26.9, and 36.2%. Statistical analysis showed that both lines of C/Sdn/EL858R tritransgenic mice had significantly less areas of lung hyperproliferative lesions than the C/EL858R bitransgenic mice at 4, 6, and 8 weeks after Dox-induction (Fig. 5B). At the 8-week time point, 3 out of 10 C/S65/EL858R tritransgenic mice and 3 out of 10 C/S669/EL858R tritransgenic mice had essentially normal lung histology or minimal hyperproliferative lesions (Fig. 5A), whereas all lungs from the 11 C/EL858R bitransgenic mice had >30% areas of hyperproliferative lesions. Moreover, lung lesions in the C/EL858R bitransgenic mice displayed a more aggressive phenotype with the appearance of multifocal compact masses of adenomas and adenocarcinomas (Fig. 5A).

### Gab1 tyrosine phosphorylation and Shp2 binding to Gab1 are inhibited in the C/Sdn/CL858R tritransgenic mice

Shp2CSDA inhibited EGFR^L858R^-induced Erk1/2 activation in the lungs of transgenic mice (Fig. 4 and Supplementary Fig. 3). Previous work has established that Shp2 binding to Gab1 (or Gab2) is essential for Shp2 to mediate Erk1/2 activation [2, 3, 5, 26].

**Figure 6:**
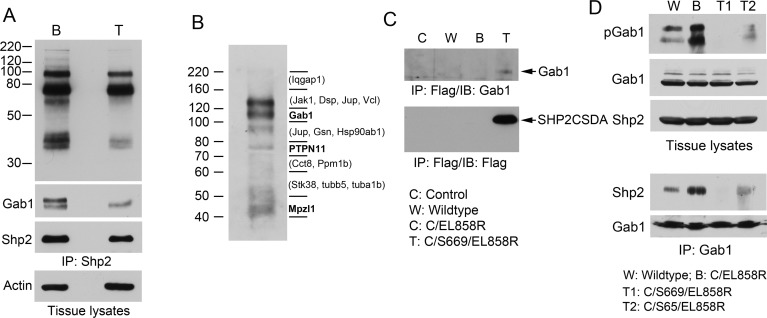
Analysis of Gab1 tyrosine phosphorylation and association with Shp2 in transgenic mouse lung A, lung tissue lysates (4 mg/each) of C/EL858R (B) and C/S669/EL858R (T) mice induced with Dox diet for 6 weeks were immunoprecipitated with anti-Shp2. Portions of immunoprecipitates (1/5, 1/5, 1/20) were analyzed by immunoblots with antibodies to phosphotyrosine (PY20), Gab1, and Shp2, respectively. Ten μg of tissue lysates were also analyzed by immunoblot with an antibody to actin. B, Lung tissue lysates from C/S669/EL858R mice induced with Dox diet for 4 weeks were immunoprecipitated with an anti-Flag antibody. One-tenth of immunoprecipitates were analyzed by immunoblotting with an anti-phosphotyrosine antibody (PY20). The rest of immunoprecipitates were separated on a SDS-polyacrylamide gel in the same experiment and gel slices were subjected to trypsin-digestion and protein identification by LC/MS/MS. Bold letters indicate tyrosine phosphorylation residues have been identified on these proteins. Proteins in parentheses are those that known to be tyrosine phosphorylated (based on www.phosphosite.org) that were detected in the indicated gel slices, but their tyrosine-phosphorylated tryptic peptides were not detected in our experiment. C, Mouse lung tissue lysates from indicated genotypes fed with Dox diet for 4 weeks were immunoprecipitated with the anti-Flag antibody and then immunoblotted with indicated antibodies. D, Transgenic mice of indicated genotypes were fed with Dox diet for 4 weeks. Lung tissue lysates were analyzed by immunoblotting with indicated antibodies (top panels). Gab1 was immunoprecipitated from these lung tissues and immunoblotted with antibodies to Shp2 or Gab1 (lower panels).

Shp2 from lung of Dox-induced C/S669/EL858R tritransgenic mice appeared to have less associated tyrosine phosphorylated proteins and Gab1 than that from Dox-fed C/EL858R bitransgenic mice (Fig. 6A). To determine if Shp2CSDA bound to Gab1 or Gab2 in the lungs of our tritransgenic mice, we immunoprecipitated the Flag-tagged Shp2CSDA from lung tissues of Dox-induced C/S669/EL858R mice. Immunoprecipitates were separated on gels and processed for immunoblotting analysis with an anti-phosphotyrosine antibody or proteomic analysis (Fig. 6B). Tyrosine phosphorylated Gab1 (on Y659) was detected in one of the strong reactive bands to the anti-phosphotyrosine antibody. Gab2 was not found in the Shp2CSDA immunoprecipitates by the proteomic analysis, possibly because Gab2 is not expressed in mouse lung epithelial cells. Other tyrosine-phosphorylated proteins detected were Shp2 (on Y62 and Y580 of the human Shp2CSDA) and Mpzl1 (on Y263). No mouse-specific Shp2 tryptic peptide was detected in the Flag-tagged Shp2CSDA immunoprecipitates by the mass spectrometric analysis (Supplementary Fig. 5). In a separate experiment, we confirmed co-immunoprecipitation of Shp2CSDA and Gab1 in lung tissues from Dox-induced tritransgenic mice by immunoblotting the Shp2CSDA immunoprecipitates with an anti-Gab1 antibody, although the amount of Gab1 protein co-immunoprecipitated with Shp2CSDA was low (Fig. 6C).

We next compared Gab1 tyrosine phosphorylation at the Shp2 binding site (pY627) and Gab1-bound Shp2 in lung tissues from the wildtype, C/EL858R, and C/Sdn/CL858R mice Dox-induced for 4 weeks. Elevated pGab1 (Y627) and Gab1-bound Shp2 were observed in the lung tissue from C/EL858R bitransgenic mice (Fig. 6D). Tissues from both C/S65/EL858R and C/S669/EL858R tritransgenic mice displayed markedly decreased pGab1 and Gab1-bound Shp2 (Fig. 6D). These results indicate that Shp2CSDA not only competed with the endogenous Shp2 for Gab1 binding but also reduced Gab1 phosphorylation at the Shp2 binding site, thus preventing binding and activation of the endogenous Shp2 by EGFR^L858R^ in the lungs of tritransgenic mice.

## DISCUSSION

It was previously unclear whether Shp2 is activated by oncogenic EGFR mutants and whether inhibition of the Shp2 PTP activity can suppress lung tumorigenesis *in vivo*. In this study, we generated novel transgenic mice containing a Dox-inducible PTP-defective Shp2CSDA. We found that endogenous Shp2 is activated in the lung tissues of transgenic mice *in vivo* by the EGFR^L858R^ mutant. Importantly, expression of the dominant-negative Shp2CSDA mutant suppressed EGFR^L858R^-induced lung adenocarcinoma in tritransgenic mice. These results suggest that Shp2 contributes to the EGFR mutant-induced lung tumorigenesis and that inhibiting the PTP activity of Shp2 can suppress EGFR mutant-induced lung adenocarcinomas.

In human lung cancer cell lines harboring either the wildtype or mutant EGFR, it was reported that Shp2 and Erk1/2 were less active in cells harboring mutant EGFR [27, 28]. Shp2 Y542 phosphorylation was used as the marker of Shp2 activation in these studied. However, it has reported that EGFR does not phosphorylate Shp2 C-terminal tyrosine residues well and the potential Y542 phosphorylation mediated docking function only plays a peripheral, if any, role in EGFR signaling [29]. Moreover, constitutive Erk1/2 activation by mutant EGFR could result in feedback inhibition that may contribute to a lower level of constitutive or transient peak pErk1/2. It is possible that other genetic alternations in the few wildtype EGFR cell lines also contribute to Erk1/2 activation in these cells. In fact, the difference in Erk activation in MEF cells transfected with wildtype and mutant EGFR was small [27].

Gab1-Shp2 binding activates Shp2 [2]. Constitutive Gab1-Shp2 binding in human lung adenocarcinoma cells harboring mutant EGFR was observed in a previous study [28] and in this study (Fig. 1). Moreover, constitutive pErk1/2 was readily detectable in these cells that carry mutant EGFR. Using Shp2 siRNAs, Dox-inducible shRNAs, Shp2 binding defective Gab1 mutant, and PTP-inactive Shp2 mutant, we have provided evidence that inhibition of Gab1-Shp2 interaction and Shp2 PTP activity suppress Erk1/2 activation in the HCC827 lung adenocarcinoma cells that harbor a mutant EGFR. Although Shp2 knockdown and a Shp2 PTP inhibitor suppress cell proliferation and pErk1/2, it is unlikely that inhibition of Erk1/2 is the only mechanism that Shp2 mediates cell proliferation or tumorigenesis. Nevertheless, pErk1/2 level provides a marker suitable for monitoring Shp2 inhibition in many cases.

We found previously that the constitutively active Shp2^E76K^ mutant increased Gab1 tyrosine phosphorylation in the lungs of transgenic mice and in cell lines via a mechanism involving Src family kinases [17], suggesting that the active Shp2 exerts a positive feedback loop to regulate its binding to Gab1. Data in this study show that the PTP-defective Shp2CSDA mutant suppresses Gab1 tyrosine phosphorylation and Src family kinases in the lung of tritransgenic mice. This finding provides another line of evidence to reinforce the notion that Shp2 controls a positive feedback loop to regulate its own activity. In the case of GOF active Shp2 mutants, this autoregulatory loop is essential for Gab1/Gab2 phosphorylation necessary for the oncogenic activity, because an active Shp2 PTP needs to dock to Gab1/Gab2 to exert its function. Since the endogenous mouse Shp2 in the lungs must dock to Gab1 in order to mediate mutant EGFR signaling, our finding that Shp2CSDA suppresses Gab1 tyrosine phosphorylation and Gab1-Shp2 binding provides a mechanistic insight into how Shp2CSDA exerts a dominant negative effect on the endogenous Shp2. Consistent with regulation of SFKs by Shp2, our data show that active SFK levels were decreased in tritransgenic mice expressing Shp2CSDA, implicating SFKs in the Shp2 autoregulatory loop in these mice, although this remains to be verified experimentally.

Our transgenic animal study represents a significant advance over previous subcutaneous tumor xenograft experiments with breast and lung cancer cells [6, 7]. First, Shp2 knockdown in tumor cells depletes the entire Shp2 protein. Besides the PTP activity, Shp2 contains SH2 domains and two Grb2-binding tyrosine phosphorylation sites. When Shp2 is knocked down, all three functional regions of Shp2 are lost. These include not only the PTP activity but also the potential SH2 domain competition and Grb2-binding activity of Shp2 C-terminal phosphorylation sites. Thus, Shp2 knockdown may not reflect the true phenotype when the Shp2 PTP activity is inhibited by a drug because the small molecular PTP inhibitor only suppresses Shp2 PTP activity while leaving the SH2 domains and tyrosine phosphorylation sites intact. By analogy, a Shp2 knockout mouse model would have the same limitation. In contrast, Shp2CSDA only affects the PTP activity and thus is a better model of Shp2 PTP inhibition. Second, previous studies measured subcutaneous tumor xenografts not grown in their natural environments in immunocompetent hosts. Our study is the first evaluation of Shp2 inhibition in a PTK oncogene-driven tumor development model in genetically engineered animals.

A major mechanism of drug resistance to EGFR TKIs in lung adenocarcinoma is reactivation of the mutant EGFR PTK activity. Moreover, *de novo* resistance to EGFR TKIs has also been associated with constitutively active EGFR PTK activity. Under these circumstances, our data suggest that the Gab1-Shp2 pathway is activated in these EGFR inhibitor-resistant lung adenocarcinoma cells and promotes tumor growth. Increasing evidence suggests that PTK-PTP cooperate to promote human cancer development and PTP inhibitors are being developed as potential anti-cancer drugs. Our finding that the PTP-defective Shp2CSDA suppresses EGFR^L858R^-induced lung adenocarcinoma in transgenic mice suggests that Shp2 PTP is a potential target for therapeutic development in EGFR-driven lung adenocarcinoma. Consistent with previous studies in cell cultures of lung cancer cells, we observed elevated pAkt and pStat3 in the lungs of transgenic mice when EGFR^L858R^ was expressed. While inhibition of Shp2 had little effect on pAkt and pStat3 in our mouse model, it is possible but remains to be tested that combining Shp2 inhibition with blockage of the phosphoinoside-3-kinase-Akt pathway and/or Stat3 signaling may be a more effective therapy for EGFR mutant-associated NSCLC that develop resistance to the first-line EGFR PTK inhibitor therapy.

## MATERIALS AND METHODS

### Reagents

On-target Smart pool Shp2-specific and non-silencing control siRNAs were from Dharmacon. Shp2 shRNAs were described [6]. Gab1FF, Shp2CSDA, and HA-Erk2 plasmids used in transfected experiments in cell cultures have been described [2, 3, 5]. Erlotinib was from LC Chemicals. WZ4002 was from Selleck. SPI-112Me was synthesized as described [11]. Antibodies to Shp2, Erk1/2, phospho-Erk1/2 (pErk1/2, T202/Y204), Gab1, Akt, c-Myc, and β-actin were obtained from Santa Cruz Biotechnology. Antibodies to Flag (rabbit), phospho-Gab1 (pGab1, Y627), phospho-Akt (pAkt, S473), and phospho-Src (pSrc, Y416) were from Cell Signaling Technology. Src antibody was from Calbiochem. The anti-Flag M2 monoclonal antibody was from Sigma.

### Cell cultures, transfection, and cell proliferation assay

Cell lines have been maintained in a central repository at the Moffitt Cancer Center since 2008. All cell lines in the Moffitt repository had been authenticated by STR analysis (ACTG Inc, Wheeling, IL) as of September 2010, and all cells had been routinely tested and were negative for mycoplasma (PlasmoTest, InvivoGen, San Diego, CA). HCC827 and H1975 lung carcinoma cells were cultured in RPMI1640 plus 10% fetal bovine serum (FBS) at 37°C in 5% CO_2_. To examine the effect of siRNA on cell proliferation, HCC827 and H1975 cells were plated in 96-well plates (2,000 cells/well) in RPMI1640/10%FBS for 24 h. Cells were transfected with 25 nM PTPN11 on-target smart pool siRNAs or non-silencing control siRNA using Lipofectamine 2000 (Life Technologies) for 4 h. After 6 days, viable cells were measured using CellTiter-Glo reagent (Promega). For immunoblotting analysis, HCC827 and H1975 cells were plated in 12-well plates (20,000 cells/well) in RPMI1640/10%FBS for 24 h before transfection with 25 nM siRNAs for 6 h as above. Cell lysates were prepared and analyzed 3 days after transfection. For SPI-112Me treatment, HCC827 and H1975 cells were plated in 96-well plates (1,000 cells/well) in RPMI1640/10%FBS for 24 h and then incubated with SPI-112Me or solvent control for 6 days before analyzing relative cell numbers using CellTiter-Glo reagent.

Transfection of HCC827 cells with Gab1 and Shp2 mutants were performed in 6-cm plates containing overnight cultures of 7×10^5^ cells/plate. Each plate of cells were transfected with 2 μg HA-Erk2 plasmid plus 2 μg of either pCDNA3.1 vector, pCDNA3.1-Gab1FF, or pCDNA3.1-Shp2CSDA using lipofectamine 2000. Forty-eight h after transfection, cells were serum-started for 18 h and stimulated with EGF (2 ng/ml, 10min) or left untreated. HA-Erk2 was immunoprecipitated and analyzed by immunoblotting as described [5].

### Immunoblotting and immunoprecipitation

Frozen tissues and cells were lysed for 1 h at 4 °C using lysis buffer (50 mM Tris-HCl, pH 7.5, 150 mM NaCl, 1 mM EDTA, 1 mM EGTA, 25 mM NaF, 5 mM Na_4_P_2_O_7_, 1 mM dithiothreitol, 1 mM Na_3_VO_4_, 100 μg/ml of phenylmethylsulfonyl fluoride, 2 μg/ml leupeptin, 2 μg/ml aprotinin, and 1% Triton X-100). Equal amounts of proteins from cleared lysates were separated by 10% SDS-polyacrylamide gels and transferred to nitrocellulose filters for immunoblotting. Flag-tagged Shp2CSDA was immunoprecipitated from cleared tissue lysate supernatants by using the anti-Flag M2 antibody. Gab1 was immunoprecipitated using Gab1 mouse antibody. Immunoblotting was performed as described previously [6, 30-32].

### Transgenic mice

A Cre recombinase-mediated cassette exchange (RMCE)-capable plasmid L3/L2-tetO (Fig. 3A) was constructed [17]. cDNA encoding Flag-tagged, C459S/D425A mutations of human Shp2 (Shp2CSDA) [5] was then subcloned into the *EcoR*V site between the tetO and polyA sequences in L3/L2-tetO to create the L3/L2-tetO-Shp2CSDA plasmid (Fig. 3A). To generate tetO-Shp2CSDA transgenic mice, the 5.8 kb *BssH*II fragment of L3/L2-tetO-Shp2CSDA transgene was isolated by agarose gel electrophoresis followed by EluTrap electroelution and EluTip purification (Whatman). Ethanol precipitated DNA was resuspended in sterile microinjection buffer (10 mM Tris-HCl, 0.1 mM EDTA, pH 7.5) and microinjected at 3 ng/μl into 0.5 dpc fertilized FVB/N zygotes. Zygotes were surgically implanted into the oviducts of 0.5 dpc pseudopregnant CD-1 females for development. Offspring were tail biopsied at weaning and genomic DNA screened by PCR to identify transgenic lines. Genotyping of tetO-Shp2CSDA transgenic mice was performed using the GoTaq® Hot Start Green Master Mix (Promega) and the following primers: SHP2T1, 5′-AGACGCCATCCACGCTGTTTTGAC-3′ and SHP2T2, 5′-TCTCTTTTAATTGCCCGTGATGTT-3′. The protocol for a 25 μl PCR reaction was: 4 min denaturation at 94 ^o^C, 35 cycles of 94 ^o^C for 30 sec, 57^o^C for 30 sec, 72 ^o^C for 30 sec with a final extension step of 72 ^o^C for 4 min, which yields a 450-bp PCR fragment.

CCSP-rtTA transgenic mice (on inbred FVB/N background) [25] were provided by Dr. Jeffrey A. Whitsett. TetO-EGFR^L858R^ transgenic mice (in B6;CBA) [19] were obtained from the NCI-Frederick Mouse Repository. The CCSP-rtTA transgenic mice were genotyped as described [25]. The tetO-EGFR^L858R^ transgenic mice were genotyped as described [19].

Animals were maintained in specific pathogen-free housing conditions. To activate the transactivating function of the rtTA protein, mice were fed with rodent chow containing 200 mg/kg doxycycline (Dox diet, Bio-Serv). Animal studies and care were approved by the Institutional Animal Care and Use Committee of the University of South Florida and followed institutional and national guidelines.

### RT-PCR analysis of Shp2CSDA mRNA

RNA was extracted from frozen tissues using Trizol reagent (Life Technologies). Samples were treated with DNase I to avoid DNA contamination and RT-PCR was performed using the SuperScript One-Step RT-PCR Platinum Taq system (Life Technologies) with the following primers: SHP2F1: 5′-GGTTGGACAAGGGAATACGG-3′ and SHP2R2: 5′-AGGGCTCTGATCTCCACTCG-3′. The protocol for a 50 μl RT-PCR reaction was: 30 min cDNA synthesis at 55 ^o^C, 4 min denaturation at 94 ^o^C then 35 cycles of 94 ^o^C for 30 sec, 57 ^o^C for 30 sec, then 72 ^o^C for 30 sec with a final extension step of 72^o^C for 4 min which yields a 462-bp fragment.

### Histological examination

After euthanasia, mouse lungs were flushed twice with 10 ml PBS, insufflated with 10% buffered formalin, and placed with the frontal side of the lungs oriented towards the bottom of embedding cassettes. After overnight fixation with formalin at room temperature, paraffin blocks were prepared using standard procedures by the Tissue Core at the Moffitt Cancer Center. Three sections (4 μm thick) were cut 25 μm apart. These tissue sections were stained with hematoxylin and eosin (H&E) for histological examination and analysis of hyperproliferative lesions.

After histological examination by two pathologists, H&E stain slides were scanned using a ScanScope XT (Aperio) with a 20x/0.8NA objective lens at a rate of 5 minutes per slide via Basler tri-linear-array to acquire whole slide images. Genie® v1 histology pattern recognition software (Aperio) was used to segment hyperproliferative lesions from other lung tissue areas and background. This was accomplished using the Aperio Nuclear® v9.1 algorithm with a training set of 400 iterations and pathologist quality control. The optimized thresholds were: Averaging radius = 1 μm; Segmentation Type = 2 Cytoplasmic Rejection; Threshold Type = 1- Edge Threshold with weighted Trimming; Min/Max Nuc Size = 25 μm^2^/1000000 μm^2^/; RGB stain = 0/0.64307/0.31756; Positive RGB OD = 0.244583/0.509334/0.825081. The resultant hyperproliferative areas were calculated as a percentage of lung tissue areas.

### Proteomic analysis

Protein identification by mass spectrometry was performed by the Proteomics Core of the Moffitt Cancer Center using standard procedures. Briefly, gel slices were treated with TCEP and iodoacetamide to reduce and alkylate proteins. After in-gel trypsin digestion, peptides were extracted and concentrated. A nanoflow ultra high performance liquid chromatograph (RSLC, Dionex, Sunnyvale, CA) coupled to an electrospray ion trap mass spectrometer (LTQ-Orbitrap, Thermo, San Jose, CA) was used for tandem mass spectrometry peptide sequencing analysis. Five tandem mass spectra were collected in a data-dependent manner following each survey scan. The MS scans were performed in Orbitrap to obtain accurate peptide mass measurement and the MS/MS scans were performed in linear ion trap using 60 second exclusion for previously sampled peptide peaks. Mascot and Sequest searches were performed against the Swiss-Prot mouse and human databases. Dynamic modifications included carbamidomethylation (Cys), oxidation (Met), and phosphorylation (Ser/Thr/Tyr). Both Mascot and Sequest search results were compiled in Scaffold.

### Statistical analysis

Statistical analysis was performed using unpaired t test with Welch's correction, without assuming equal SDs. A difference in means with *p* < 0.05 was considered statistically significant.

## SUPPLEMENTARY MATERIAL, FIGURES


