# Supplementary material for: Inhibition of Shp2 suppresses mutant EGFR-induced lung tumors in transgenic mouse model of lung adenocarcinoma
Source: Oncotarget. 2015 Jan 31;6(8):6191–202. doi: 10.18632/oncotarget.3356 (PMC4467431; doi:10.18632/oncotarget.3356)
Supplement: Supplementary file 1 [file oncotarget-06-6191-s001.pdf]

Inhibition of Shp2 suppresses mutant EGFR-induced lung tumors in transgenic mouse model of lung adenocarcinoma

Supplementary Material

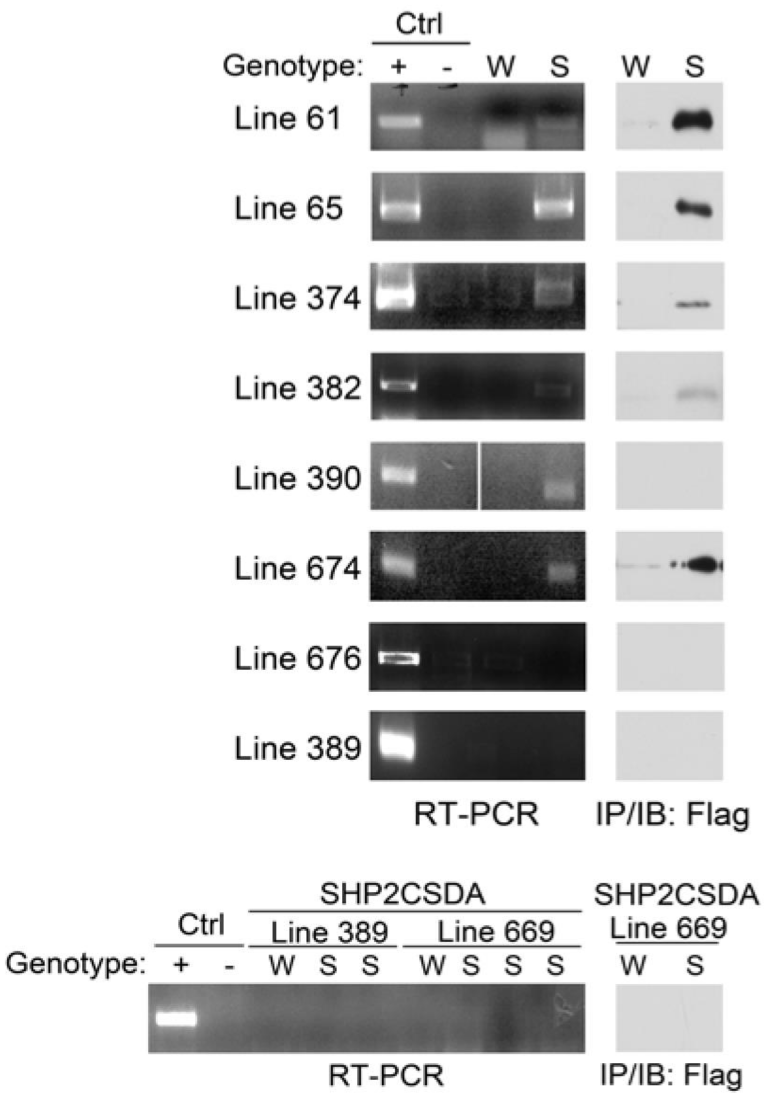

Supplementary Figure 1: Characterization of tetO-Shp2CSDA founder lines. Lung tissues from the wildtype (-) or tetO-Shp2CSDA founder lines (+) were analyzed by RT-PCR or immunoprecipitation-immunoblotting with anti-Flag tag antibodies to analyze mRNA and protein expression in the lung of these mice.

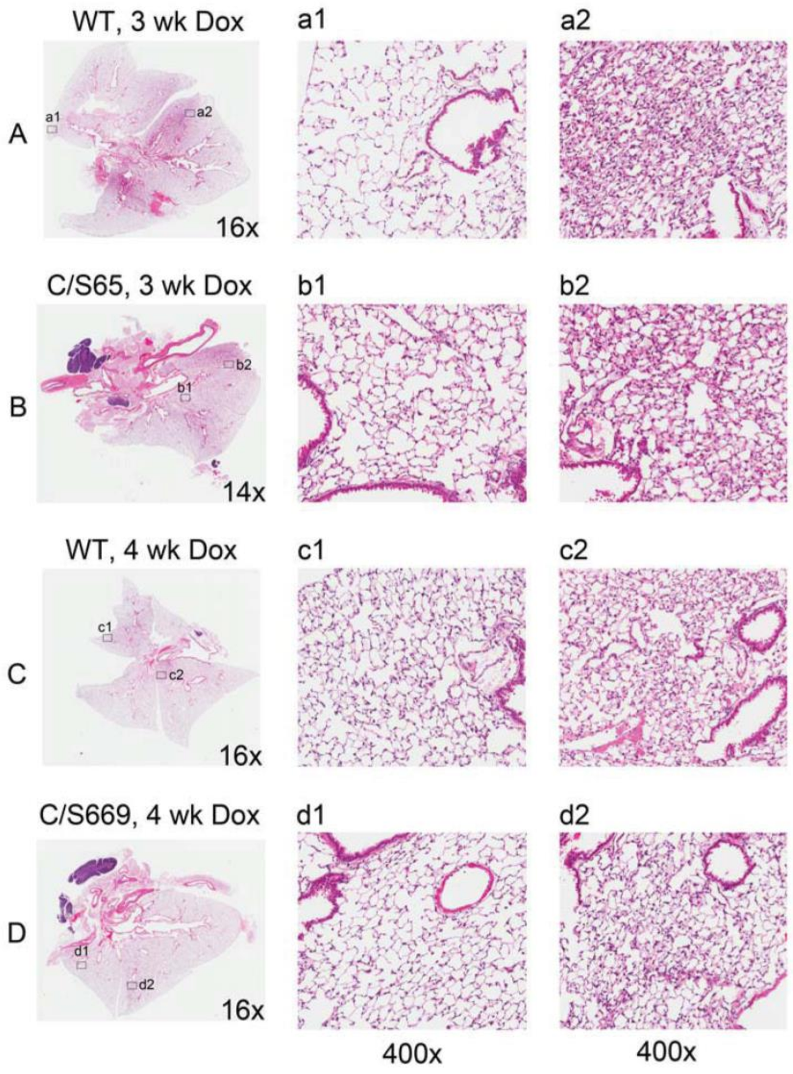

Supplementary Figure 2: H&E stained lung tissue sections. Wildtype and C/S65 or C/S669 bitransgenic mice were fed with Dox diet for 3 to 4 weeks as indicated. Lung were flushed twice with 10 ml PBS and insufflated with 10% buffered formalin. Formalin-fixed, paraffin-embedded lung tissue sections (4 μm thick) were stained with H&E. The middle and right panels are a higher magnification (400x) of the boxed areas. a1-d1, areas show normal alveoli with no pathologic changes. a2-d2, areas show foci of lung parenchyma with atelectasis (likely due to insufficient lung insufflation during organ processing) and a few alveolar macrophages.

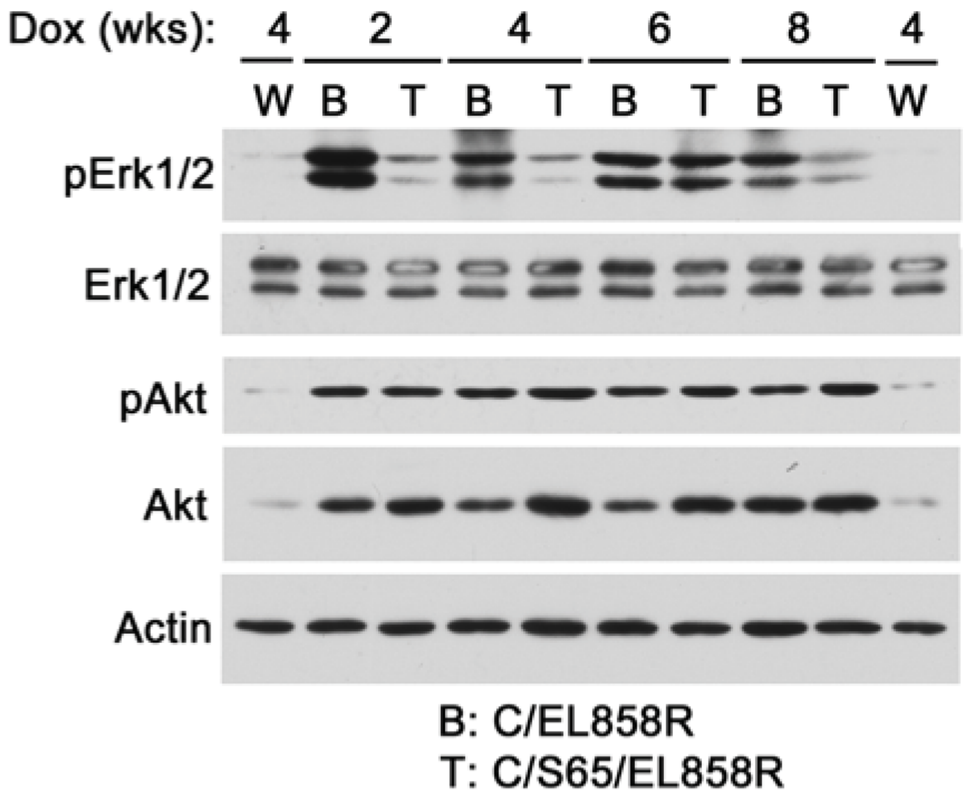

Supplementary Figure 3: Analysis of EGFR^{L858R}-activated Erk1/2 and Akt in transgenic mice. C/EL858R bitransgenic and C/S65/EL858R tritransgenic mice were fed with Dox diet for 2-8 weeks. Lung tissue samples were analyzed by immunoblotting with antibodies indicated in each panel.

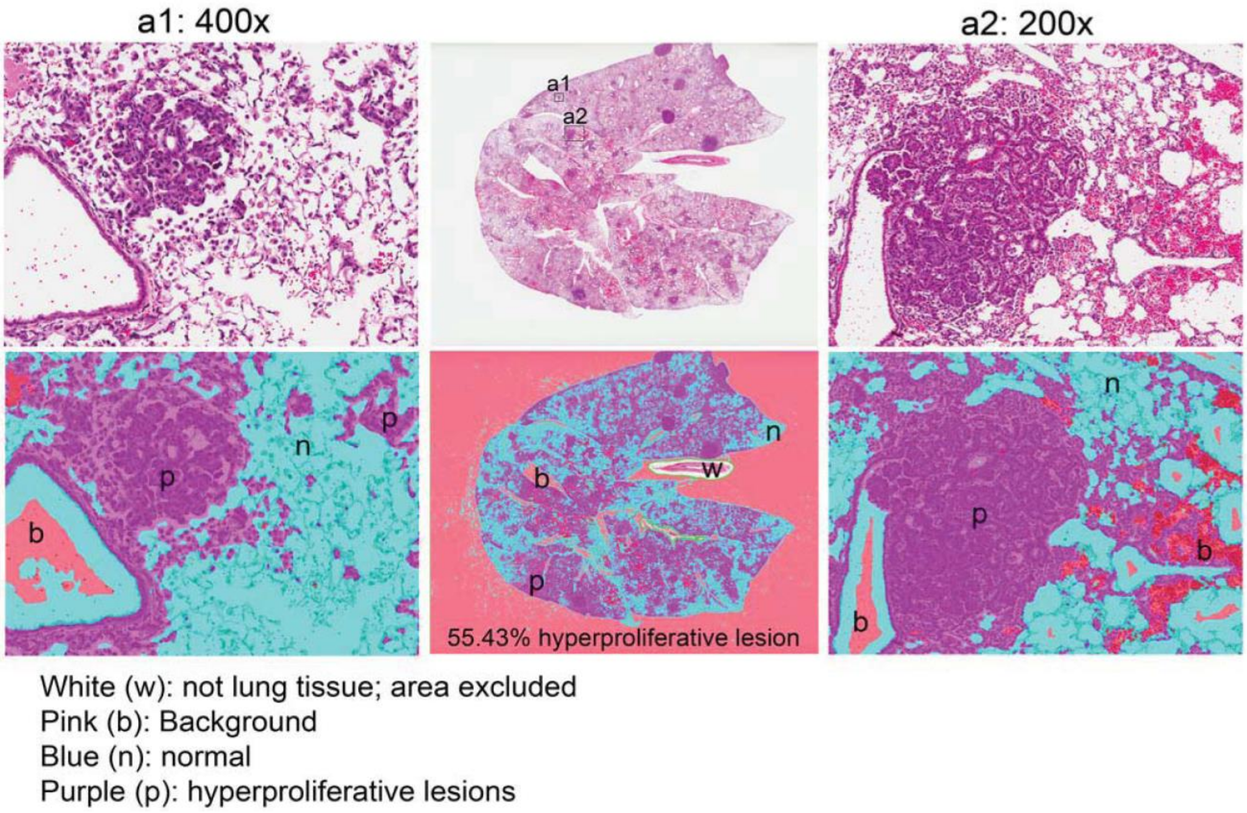

Supplementary Figure 4: Illustration of histology patterns recognized in the lung tissue section by the pattern recognition algorithm. The image of a H&E stained section was analyzed by the Genie® histology pattern recognition program (Aperio). Upper panels: H&E stain. Middle panel, whole lung section; side panels, higher magnifications of the boxed areas. Lower panels: tissue histology recognized by the algorithm. The optimized thresholds were obtained from a training set of 400 iterations. Airway epithelia were recognized as hyperproliferative lesions. Areas other than lung tissue and background areas were excluded from calculation. The percentage of hyperproliferative lesion was calculated by dividing hyperproliferative lesions with (normal + hyperproliferative lesions).

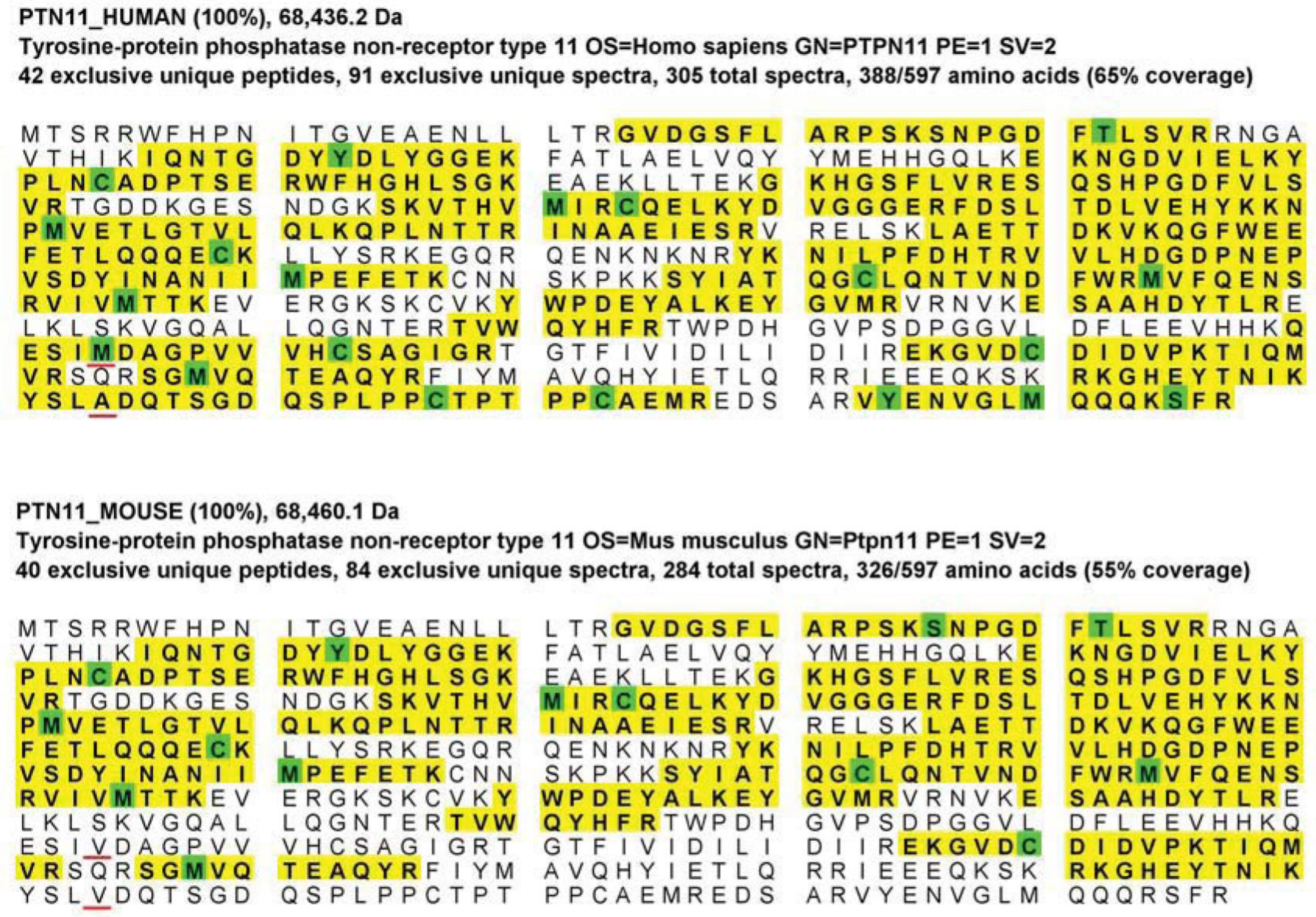

Supplementary Figure 5: No mouse-specific Shp2 tryptic peptide was identified in Flag- Shp2CSDA immunoprecipitates from mouse lung tissues. Shp2CSDA immunoprecipitates were analyzed by LC/MS/MS for protein identification. Shp2 tryptic peptides detected by the proteomic analysis are highlighted in yellow. Note that mouse-specific Shp2 peptides containing V454 and V554 were not found, whereas the corresponding human Shp2 peptides containing M454 and A554 were detected. Residues highlighted in green are modified residues.
